# Stigma and fear during the COVID-19 pandemic: a qualitative study on the perceptions of healthcare workers in Canada and Singapore

**DOI:** 10.3389/fpubh.2024.1490814

**Published:** 2025-01-23

**Authors:** Christine Fahim, Chou Chuen Yu, Jeanette Cooper, Suvabna Theivendrampillai, Taehoon (Tom) Lee, Michelle Wai-Ki Lau, Christine Marquez, Bernard Tang, Mathews Mathew, Malika Sharma, Eric Wong, Tracey O'Sullivan, James Alvin Low, Sharon E. Straus

**Affiliations:** ^1^St. Michael's Hospital, Unity Health Toronto, Toronto, ON, Canada; ^2^Institute of Health Policy, Management, and Evaluation, University of Toronto, Toronto, ON, Canada; ^3^Geriatric Education and Research Institute Ltd, Singapore, Singapore; ^4^Institute of Policy Studies, National University of Singapore, Singapore, Singapore; ^5^Interdisciplinary School of Health Sciences, University of Ottawa, Ottawa, ON, Canada; ^6^Department of Geriatric Medicine, Khoo Teck Puat Hospital, Singapore, Singapore

**Keywords:** health personnel, SARS-CoV-2, social stigma, perceived discrimination, racism, intersectional framework, Canada, Singapore

## Abstract

**Introduction:**

We sought to explore healthcare providers (HCPs)' perceptions of and experiences with stigma during the COVID-19 pandemic in Canada and Singapore.

**Methods:**

We conducted a qualitative study (May 2020–February 2021) with HCPs in Canada and Singapore and developed a semi-structured interview guide rooted in the Health Stigma and Discrimination Framework (HSDF). We recruited participants online and through word of mouth via newsletters, blogs and social media. Participants were eligible to participate if they worked as a healthcare provider in Singapore or Canada during COVID-19. Following participant consent, data were recorded, transcribed verbatim, and coded using a framework approach. Coded data were charted into a framework matrix and used to compare themes in each country.

**Results:**

We conducted 51 interviews (23 in Canada; 28 in Singapore). HCPs perceived that patient fears coupled with mistrust of the health system impacted health behaviors. HCPs reported discrimination and stigmatization of population subsets. In Singapore, this included Chinese tourists and migrant workers and in Canada, this included people of Chinese ethnicity and people experiencing homelessness. This stigma was often attributed to pre-existing prejudices including perceptions that these populations were at increased risk of COVID-19 or not adhering to public health recommendations. HCPs feared spreading COVID-19 to family, peers and patients, often resulting in participants choosing to isolate from social circles. HCPs in both countries experienced occupation-based stigma, including stigma related to public health practices (masking, testing); in Canada, this intersected with race-based prejudice for participants of Chinese ethnicity. HCPs in both samples witnessed and experienced stigmatizing behavior; some participants also experienced discrimination.

**Conclusions:**

Secondary stigma related to occupation was experienced by HCPs during COVID-19. HCPs experienced intersecting stigma based on race/ethnicity and observed stigmatization of marginalized patient populations. Most themes were consistent across Canada and Singapore. Strategies to mitigate COVID-19 related stigma toward HCPs and at-risk patient populations are warranted.

## 1 Introduction

Stigmatization toward healthcare professionals (HCPs) caring for patients living with infectious diseases including HIV and AIDS, Ebola, Severe Acute Respiratory Syndrome (SARS), and tuberculosis has been widely reported (1–3). Authors of a recent systematic review estimated the pooled prevalence of infectious disease stigma across patients, communities and HCPs at 34% and highlighted infectious disease stigma as a significant public health concern (4). The impact of infectious disease stigma can lead to discrimination, social isolation, and negative impacts on mental health (5–8). Stigmatization of HCPs providing care to patients living with infectious diseases not only results in significant harm to the HCP, but can also be a barrier to a resilient frontline clinical response during large outbreaks (9).

Research has demonstrated similar patterns of stigmatization toward people who were sick with COVID-19 and toward the HCPs who cared for them (1–3) In Canada, the onset of the pandemic led to an emergence of race-based prejudice and discrimination toward Chinese-Canadians and individuals appearing to be of East Asian ethnicity. These narratives were further perpetuated by the media (10), including early referrals to SARS-CoV-2 as the “Wuhan virus” or the “China virus” by US and Canadian news outlets (11). At the time, Singapore had the highest number of COVID-19 cases outside of China (12). The country was also host to one of the most popular destinations for Chinese tourists, particularly during the Lunar New Year (13) (January 2020, when SARS-CoV-2 was sequenced in Wuhan and when the WHO declared emergence of the “novel coronavirus”) (14).

In this context, we aimed to conduct a cross-country comparative study to determine whether the race-based stigmas observed in Canada were also prevalent in Singapore (where >70% of individuals are ethnically Chinese) and to explore whether other intersecting stigmas were present and how these differed by country context. To address these aims, we conducted a series of research studies that explored public, healthcare provider, and policymaker perceptions of stigma during the COVID-19 pandemic (11, 15, 16). In this study, we report the findings of our interviews with HCPs in the two countries. We used the Health Stigma and Discrimination Framework (HSDF) (17) to explore HCPs' perceptions of stigma during the COVID-19 pandemic with a focus on describing, comparing and contrasting HCPs' personal experiences of fear and stigma during the COVID-19 pandemic, in two countries. We also aimed to explore HCPs' perceptions and consequences of stigmatization on patient populations.

## 2 Methods

To enhance the completeness and transparency of our study, we report our methods using the Consolidated criteria for reporting qualitative research (COREQ) checklist (18) (see Additional File 1).

### 2.1 Setting

Our study was conducted in the province of Ontario, Canada and the city-state of Singapore. Pre-existing collaborations between our research teams at Unity Health Toronto in Canada and the Geriatric Education and Research Institute in Singapore facilitated the rapid initiation of this study during the pandemic.

### 2.2 Study design

Our study design was rooted in phenomenology, whereby we aimed to explore understand HCP's observances and experiences of stigmatization during the COVID-19 pandemic. We analyzed our data using the Framework approach, which provides a pragmatic and flexible qualitative methodology that allows for charting and comparisons of “cases” across contexts. Interviews were conducted and analyzed independently by each country team; key themes were subsequently compared and contrasted across the teams (19). The teams met iteratively throughout the project period to discuss decisions related to recruitment, data collection and analysis.

### 2.3 Participant recruitment

Prior to initiation, this study was approved by the Unity Health Toronto Research Ethics Board (study number 20-092) in Canada and the National Healthcare Group Domain Specific Review Board (reference number 2020/00582) in Singapore.

We used convenience and snowball sampling recruitment strategies. Participants were recruited through advertisements disseminated online and through word of mouth via our health and professional networks, newsletters, blogs and social media. In Singapore, the official language of communication in the healthcare setting is English and interviews were offered in English. Interviews in Ontario, Canada were offered in the official languages of English and French, as well as Mandarin and Cantonese. The Canadian research team collaborated with the Chinese Canadian National Council Toronto Chapter (CCNCTO), who championed awareness and activism campaigns to highlight the prevalence of Anti-Asian racism during the COVID-19 pandemic. The CCNCTO supported translation of our interview guides to Mandarin and Cantonese, which are some of the most commonly spoken languages in Canada (20).

In Canada, prospective participants were invited to complete a demographic questionnaire offered in English, Cantonese and Mandarin through Qualtrics, a secure, online platform (21). The questionnaire was used to inquire about participants' gender, age, occupation, region of residence and education. Questionnaire completion was voluntary. In Singapore, prospective participants were screened via phone call. Eligible participants were (a) individuals working as a HCP at time of interview (includes individuals providing clinical care within a clinical care setting), (b) English speaking (for Singapore participants) or English, French, Mandarin or Cantonese speaking (for Canada participants), (c) able to provide informed consent, (d) living in Singapore or Canada during the pandemic, (e) aged ≥18 years (for Canada participants) or ≥21 years (for Singapore participants), and (f) able to participate in an interview via Zoom online platform or phone (22). We aimed for a minimum sample size of *n* = 13 interviews per country [following Francis' guidance of *n* = 10 with a stopping criterion of *n* = 3 for theme saturation (23)]; however, we continued interviewing until theme saturation was achieved.

### 2.4 Data collection

The HSDF by Stangl and colleagues informed interview guide development (17). The HSDF describes health stigma across a socio-ecological spectrum and aims to distinguish between stigmatized experiences (i.e., direct experiences of mistreatment or discrimination) vs. stigma practices (e.g., factors and beliefs that perpetuate stereotypes and discrimination). Recognizing that infectious disease stigmatization intersects with systemic biases that are often deeply and systemically rooted, the HSDF allows for an exploration of intersecting stigmas within the socioecological context in which they manifest (17).

Key informant interviews covered five domains of the HSDF including questions on stigma drivers and facilitators, stigma marking and experiences with stigma and discrimination (see Additional File 2 for the interview guide). The interview guide was reviewed by research group members who were not involved in its development to ensure clarity and relevance to the study objectives; modifications to study questions and language were made as necessary. Key informant interviews were conducted by teleconference or Zoom. We obtained verbal consent to participation and interview audio recording. Interviews were de-identified, and each participant was assigned a unique participant ID. Interviews ranged from 30 to 60 min. in duration, depending on the participant's availability, given COVID-19 clinical demands. Interviewers did not hold any relationships with the study participants.

### 2.5 Research team and reflexivity

Interviews in Canada were conducted by research staff (TL, BSc; CM, BSc; JC, MSc; MKL, BA) and overseen by qualitative scientists (CF, PhD; SES MD). Interviews in Singapore were conducted by research officers (MK, BSocSc; BT, BPsySc) and overseen by a research fellow (CCY, PhD). The research team included women and men of diverse ethnicities, from Canada and Singapore.

### 2.6 Data analysis and interpretation

The framework approach was used to analyze the data (19). First, data were coded inductively to allow for the emergence of themes; next, these themes were deductively coded to the HSDF (17). English interviews were transcribed using NVivo Transcription, an automated transcription service (24). Researchers (TL; CM; ST, BSc; MKL; BT) reviewed and verified the transcripts to ensure accuracy. Mandarin and Cantonese interviews were transcribed verbatim by CCNCTO and translated to English. Translated transcripts were reviewed and verified for accuracy of interpretation by a research staff member (MKL).

Interviews in Canada were double coded using NVivo 11 (25), and interviews in Singapore were single coded by a research officer using QDA software (26). A coding framework was initially developed by the Canadian research team following review of three transcripts and adapted by the Singaporean team following completion of three interviews to reflect local context and themes, as identified via the deductive coding process. As coding progressed, the framework was iteratively revised to build on, refine and improve the categories.

All data were double coded iteratively until a minimum kappa of 60% was obtained, which represents moderate agreement (27). All Canadian transcripts were double coded, where each round of coding consisted of five transcripts, and a consensus meeting was held between the primary and secondary coder after each round of coding. Research staff double coded the English interviews (TL and CM) and the Mandarin and Cantonese interviews translated to English (JC and ST). Staff turnover within the Singaporean research team resulted in one research officer single coding all the interviews (BT); interviews and coding schema were then reviewed by the research fellow (CCY).

Coded data were charted into a framework matrix. The matrix consisted of code labels and illustrative quotations. Each country team created a matrix and research staff charted coded data. The matrices were used to compare theoretical concepts and map connections between categories and phenomena, using the HSDF. Descriptive statistics for demographic data were generated. A data saturation monitoring template was also formed to track salient themes (26). A research scientist in Canada (CF) used the matrices to identify similarities and differences in identified themes, by country, and these themes were discussed with a scientist from Singapore (CCY) to ensure accuracy in interpretation.

## 3 Results

### 3.1 Demographics

We conducted 51 interviews. In Canada, 24 participants were screened; 23 met eligibility criteria and were included. In Singapore, 29 participants were screened; 28 were included. Forty-seven interviews were conducted in English (19 in Canada; 28 in Singapore), one in Mandarin and three in Cantonese. Interviews in Canada were conducted from May 2020 to October 2020; interviews in Singapore were conducted between October 2020 to February 2021.

The mean duration for the Canadian interviews was 50.8 min, and 46.2 min for the Singaporean interviews. The median participant age for both samples was 39 years, and participant age ranged between 22 and 70 years (Table 1). Singaporean participants reported having more years of experience working in healthcare, with an average of 16 years experience (range: 5–43 years). In Canada, participants on average had 11.9 years of experience (range: 1–40 years).

**Table 1:** Demographic characteristics of participants.

	**Canada (*n* = 23)**	**Singapore (*n* = 28)**
**Gender** ^*^		
Man	4 (17.39%)	11 (39.28%)
Woman	19 (82.61%)	17 (60.71%)
**Age**		
18–24	1 (4.35%)	0 (0.00%)
25–34	6 (26.08%)	12 (42.86%)
35–44	8 (34.78%)	8 (28.57%)
45–54	4 (17.39%)	4 (14.28%)
55–64	2 (8.70%)	3 (10.71%)
65–74	2 (8.70%)	1 (3.57%)
75 +	0 (0.00%)	0 (0.00%)
**Race/Ethnicity**		
	East Asian 7 (30.43%)	Chinese 16 (57.14%)
	South/Southeast Asian 4 (17.39%)	Malay 4 (14.28%)
	White/Caucasian-European 3 (13.04%)	Indian 7 (25.00%)
	White/Caucasian-North American 6 (26.08%)	Other 1 (3.57%)
	Multi-ethnic 3 (13.04%)	
**Education**		
Up to 12th grade	0 (0.00%)	3 (10.71%)
Graduated high school or equivalent	0 (0.00%)	1(3.57%)
College	3 (13.04%)	3 (10.71%)
University degree	9 (39.13%)	11 (39.28%)
Post-graduate degree	9 (39.13%)	10 (35.71%)
Did not respond	7 (30.43%)	
**Field**		
Program and patient facing supports	6 (26.09%)	N/A
Nursing	5 (21.74%)	8 (28.57%)
allied health professionals	5 (21.74%)	11 (39.28%)
Physicians	4 (17.40%)	9 (32.14%)
Preferred not to respond	3 (13.04%)	N/A
**Province**		
British Columbia	1 (4.35%)	N/A; Singapore is a city-state
Alberta	1 (4.35%)	
Manitoba	2 (8.70%)	
Ontario	18 (78.26%)	
Quebec	1 (4.35%)	
**Region**		
Large urban population center, with a population of 100,000 or more	19 (82.61%)	N/A
Medium population center, with a population between 30,000 and 99,999	2 (8.70%)	
Small population center, with a population between 1,000 and 29,99	1 (4.35%)	
Rural area, with a population < 1,000	1 (4.35%)	
**Employment status**		
Full time	14 (60.87%)	26 (92.86%)
Temporary full time	2 (8.70%)	2 (7.14%)
Part time	5 (21.74%)	
Did not answer	7 (30.43%)	

^*^Participants in our sample identified as men or women, though we were inclusive of all genders and allowed participants to self identify.

Both samples had more women than men, yet the Singaporean sample was more gender balanced (82.6% women in Canada and 60.7% women in Singapore). The Canadian sample was ethnically diverse and included 39.1% who identified as White (North American or European), 30.4% who identified as East Asian, 17.4% who identified as South or Southeast Asian and 13.1% who identified with multiple ethnicities; the Singaporean sample reflected diversity of the population (28) and included 57.4% who identified as Chinese ethnicity, 25.0% who identified as Indian, and 14.3% as Malay (Table 1). Singapore is a city-state without distinct regionality. In Canada, most of the participants were from the province of Ontario. The majority of participants worked in a large urban population center, with a population of ≥100,000.

Participants were highly educated, with the majority holding a post-graduate or bachelor's degree (Table 1). The majority of the sample were employed full time (69.6% in Canada, and 92.9% in Singapore). Participants' professions included nurses (21.7% in Canada, and 28.6% in Singapore), physicians (17.4% in Canada, and 32.1% in Singapore), program and patient facing support staff (26.1% in Canada) and other health discipline professionals (e.g., psychologist, physiotherapist, radiographer, occupational therapist; 21.7% in Canada, and 39.3% in Singapore).

### 3.2 Patient fears of contracting COVID-19 coupled with mistrust of the health system were perceived to impact health behaviors

HCPs in Canada noted difficulties managing mistrust among patients toward HCPs and challenges with convincing patients to visit hospital for care during the pandemic. In Singapore, HCPs noted patients' hesitation to get tested for COVID-19 (which was standard practice in clinics/hospitals at the time) and fears of contracting COVID-19 from a hospital, as noted in the following quotations,

“*I don't think most people are willing to go to the clinic to get swabbed. They would rather stay at home, and nurse their own sickness until they are well, and then they go out” (S23)*

“*Three of them [patients] did say they didn't want to come to the hospital because they were afraid of catching the virus. They didn't want to be within the hospital grounds” (S66)*.

In Singapore, some participants observed that patients were against using Western medicine for treating flu-like symptoms and preferred to care for themselves at home. Canadian HCPs noted an increase of patients with severe complications presenting to hospital, because they had avoided the hospital out of fear of COVID-19, as noted in the following quote,

“*All of a sudden, people with heart attacks are not presenting themselves to the hospital.” (C598)*

“*There's people who are terrified and they don't want to set foot [in clinic]. Like I said before, they don't want to set foot in here nor in the hospital and I have to convince them like it's gonna be okay” (C955)*

Other HCPs did not perceive any changes in patient health seeking behaviors during the pandemic.

#### 3.2.1 HCPs reported discrimination and stigmatization of population subsets by both patients and providers

HCPs in both countries reported observing discrimination and stigmatization of population subsets that impacted these populations' health behaviors. In Singapore, Chinese tourists and migrant workers were perceived as having higher rates of COVID-19 because locals believed these groups were more susceptible to contracting the virus by virtue of country of origin or residential arrangements. HCPs reported that these populations were fearful of coming to hospital or seeking out care when sick, out of fear of being further stigmatized or, in the case of migrant workers, out of fear of losing work. These themes are illustrated in the following participant quotations,

“*They don't want to be quarantined or they don't want to be looked upon like they are the ones who's spreading [COVID]. Especially those who come back from overseas, like China. Yeah, because initially they say that COVID come from them. So that's why as people who travel overseas, they will lie [about their travel, about their symptoms].” (S37)*.

“*The migrant workers, because I know they're here [to make] a living. Maybe initially they were concerned that they would lose their income and therefore they wouldn't come forward to seek help openly.” (S52)*.

In Canada, providers noted similar trends among people of Chinese ethnicity and toward patient populations experiencing homelessness,

“*Well, I think it's [COVID-19] put the homeless population back 50 years. I mean, people were already turning their nose up at those that would be homeless or living in shelters and I think that's just gotten worse. They had to make a temporary shelter here [downtown Toronto hospital] for people who were getting tested and I overheard other people saying, ‘well, I'm not going to go there because that's what's there. Who knows what those people have? They all have COVID.' They don't have it. We're treating everything we can and doing what we can.” (C374)*

Notably, Canadian HCPs observed race-based discrimination toward Chinese patients by both their colleagues and other patients,

“*I have found some of my colleagues' words unpleasant. They would say negative things about Chinese people. I hate that and I would refute. I told them not to judge if they didn't know the whole story. My colleagues are professionals, but they still behave like that…And although I am Chinese, they don't need to comment on China and Chinese people like that in front of me.” (C412)*

Some participants in Singapore described that over time, they and their colleagues realized that structural inequalities (cramped living conditions, access to hygiene resources) rather than non-compliance with public health measures, were driving outbreaks among these populations, as noted in the following quotations,

“*Initially that ‘oh yeah', it's their [migrant workers] fault that this has happened? Is it because, you know, they congregate in large numbers and they do not bother about social distancing and that's the reason why the virus has spread so easily among that community. But I think as time went on, we realized that this was really not the case and that it really wasn't anybody's fault, least of all the migrant workers” (S45)*

“*The foreign workers, I believe that they're not well integrated in the community for sure…COVID-19 highlighted that even further” (S66)*.

#### 3.2.2 HCPs feared spreading COVID-19 to family, peers, patients which impacted their behaviors

The majority of HCPs in both samples feared contracting COVID-19 at work. Participants feared COVID-19 infection due to the mismanagement of personal protective equipment (PPE), lack of compliance with COVID-19 protocols, transmission between coworkers, and caring for patients with COVID-19. Participants were particularly fearful of becoming sick and infecting their family members, peers, and other patients, as noted in the following quotes,

“*My fear is that if I were to get it and were to pass it on to other people. Even on an exponential level that's terrifying that one infection from you can spread to so many other people. Mostly passing on to others and getting it to a large level, especially with vulnerable people like my parents. Passing it on to them gives me quite a lot of anxiety.” (C223)*

Participants also shared concerns related to staff shortages due to illness and quarantines, as noted in the following quote,

“*[My] initial fear was I didn't know how it was spread and I have children and, you know, the other part of the fear came along because I do have a department to run and when one person calls in sick, the fear set in almost immediately…how do I function the department on a day-to-day basis?” (S63)*.

As a result, participants noted themselves becoming fearful of their own colleagues, and chose to isolate themselves whenever possible, as noted in the following quote,

“*To give you an example, I had a staff [who had a relative that had COVID]…the first question that comes through is ‘is she working with us'? because you know, the fear that comes to you is what if she's working with you and she's spreading it to somebody else?” (S63)*

As the pandemic evolved, COVID-19 protocols often required those with exposures to isolate. As a result, some HCPs hesitated to get tested out of fear of how this may be perceived by their colleagues, particularly if they became sick or had to isolate, as noted below,

“*For instance, in the early days, one of our co-workers was ill, like had a cough and had a fever. Before we had really good protocols in place, so they had to go get the test, tested negative, but for whatever reason the general consensus, and I didn't agree with this, was to isolate for two weeks, even though their test was negative. So that poor person became like a leper amongst their own co-workers who didn't want them around. So I think that that probably influenced me for sure. If it came to wanting to be tested, and I think that a lot of people feel that way. They don't want to be the ones that get COVID and be looked at like a person who's spreading the virus.” (C959)*

#### 3.2.3 Secondary stigmatization due to occupation

In Canada, some participants feared secondary or associative stigmatization, as described in the HSDF, due to their occupation of caring for those ill with COVID-19; however, an equal number did not. In Singapore, more participants reported experiencing associative stigma by working with populations perceived as high risk. For instance, one participant said,

“*When I'm wearing my uniform walking outside, people may tend to stay away from me or keep further distance away from me, I realize” (S23)*

While another noted,

“*Just because I worked in the hospital, they were uncertain that I should be with this other person, with my best friend. So I guess that's technically stigma. They just assumed because I worked at the hospital, that perhaps I wasn't safe to be around them or their children. Whereas when people ask me about work, I say I feel safer at the hospital than I do at the grocery store. That still remains true. I still feel safer at work than I feel at any grocery store.”(C329)*

Some HCPs felt stigmatized for engaging in preventive behaviors at pandemic onset, such as wearing masks or using disinfectants at work, as the following participant described,

“*Yeah just a dig, a sneering comment because they think it's silly wearing a mask. Yes. And like I say, I noticed that more because masking has been become consistent for me*.” *(C200)*

In Canada, some HCPs feared intersecting discrimination based on occupational stigma and race-based prejudice, as demonstrated in the following participant quote,

“*Quite a few of my coworkers are Asian and from different countries. A lot of them have felt that the patients have not been respectful. They have experienced xenophobia from patients.” (C908)*

“*We avoid talking about that [stigma]…I do feel the stigma because of COVID… I am Chinese so I feel there's an association that most people feel that because you're Chinese it's your fault.” (C906)*

Participants that did experience manifestations of stigma, as defined by the HSDF, reported experiences of nursing staff being told to leave their rented homes (Singapore), not being able to hail a taxi or take public transport (Singapore) or being denied service at certain pharmacies or stores (Canada).

Finally, we asked participants how they dealt with stigma and discrimination faced during the pandemic. In both samples, many HCPs highlighted how they stayed focused on doing their jobs and caring for patients, which aligns with the HSDF manifestations of resilience. In instances where they did experience stigmatization, they shared statements such as “*I ignored it*,” “*I kind of got over it*,” or “*I didn't worry about that*” and as demonstrated in the following quotation,

“*Initially, I was worried about whether people would, because we are on the front line, whether people would avoid us, because we are a high risk…but I think over time, I kind of got over it because, face it, I cannot be quitting my job just because I have to eliminate the fear. I like what I'm doing and I respect my job, so all the time I will come here [to work].” (S72)*.

Some participants in Singapore felt the community was rallying behind them and was appreciative of their work during the pandemic, as noted in the following quote,

“*I must say, I received a lot of goodie bags because of COVID-19, like the most amount of goodie bags I've received in my life” (S45)*.

This theme was not expressed by participants in Canada.

## 4 Discussion

**Figure 1:**
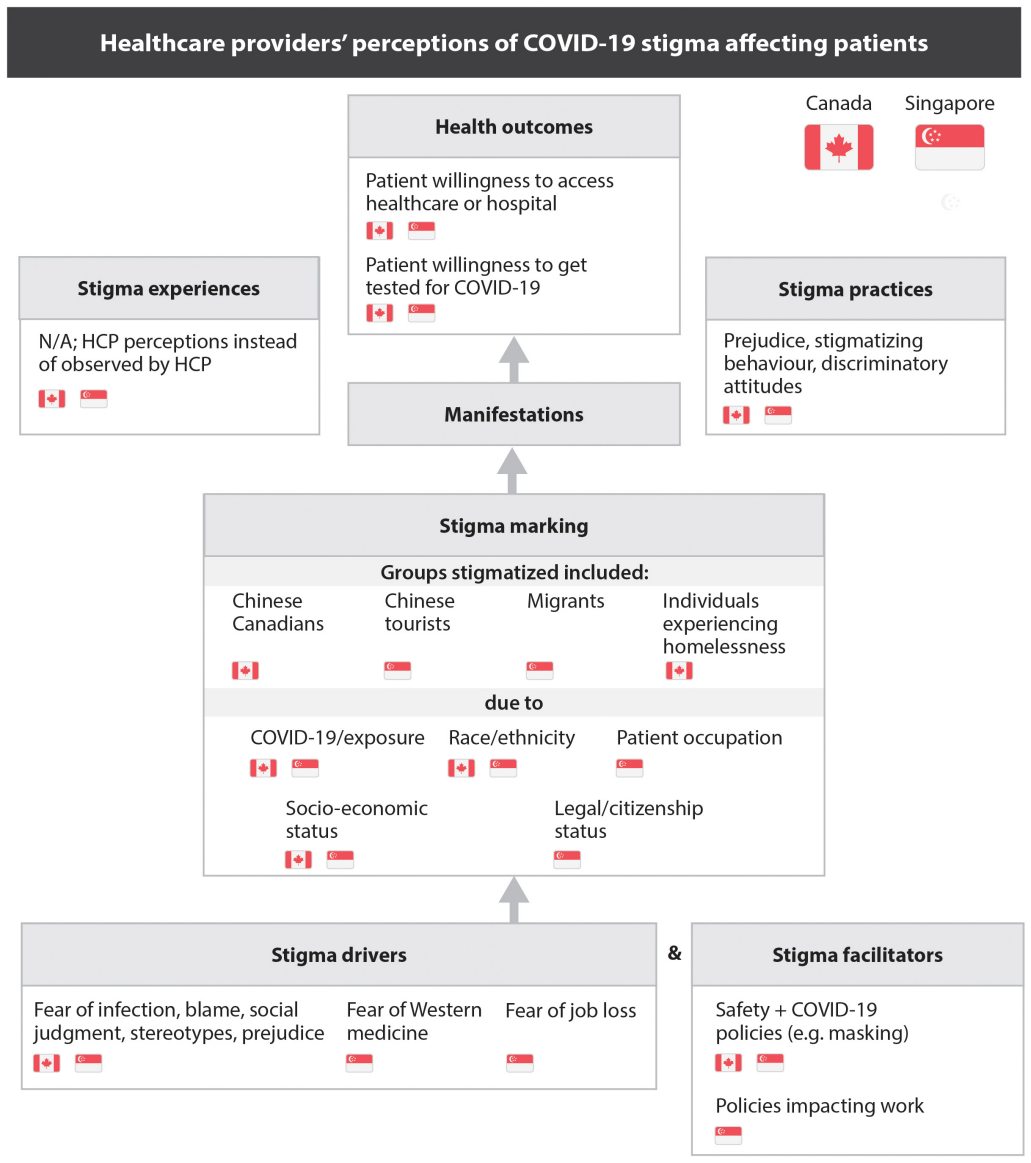
Healthcare providers' perceptions of COVID-19 stigma affecting patients.

Using the HSDF framework, we identified common themes across Canada and Singapore. HCPs perceived that stigma toward patients stemmed from the stigma drivers of fearing COVID-19 infection coupled with stereotypes, prejudice and blame toward population subsets. In both countries, individuals of Chinese ethnicity and Chinese tourists were targets of this stigma. In Singapore, migrant and temporary workers were perceived as having higher probability of having COVID-19, while in Canada, HCPs perceived an intensification of prejudice toward unhoused patients. Stigma marking for patients in both countries was attributed to race, and compounded by socioeconomic status in Canada, and occupation or legal status in Singapore. HCPs observed discriminatory behaviors toward these patient populations by both patients and colleagues. They reported that patients avoided seeking care to evade further stigmatization, with Canadian HCPs noting delayed presentations of advanced symptoms (Figure 1).

**Figure 2:**
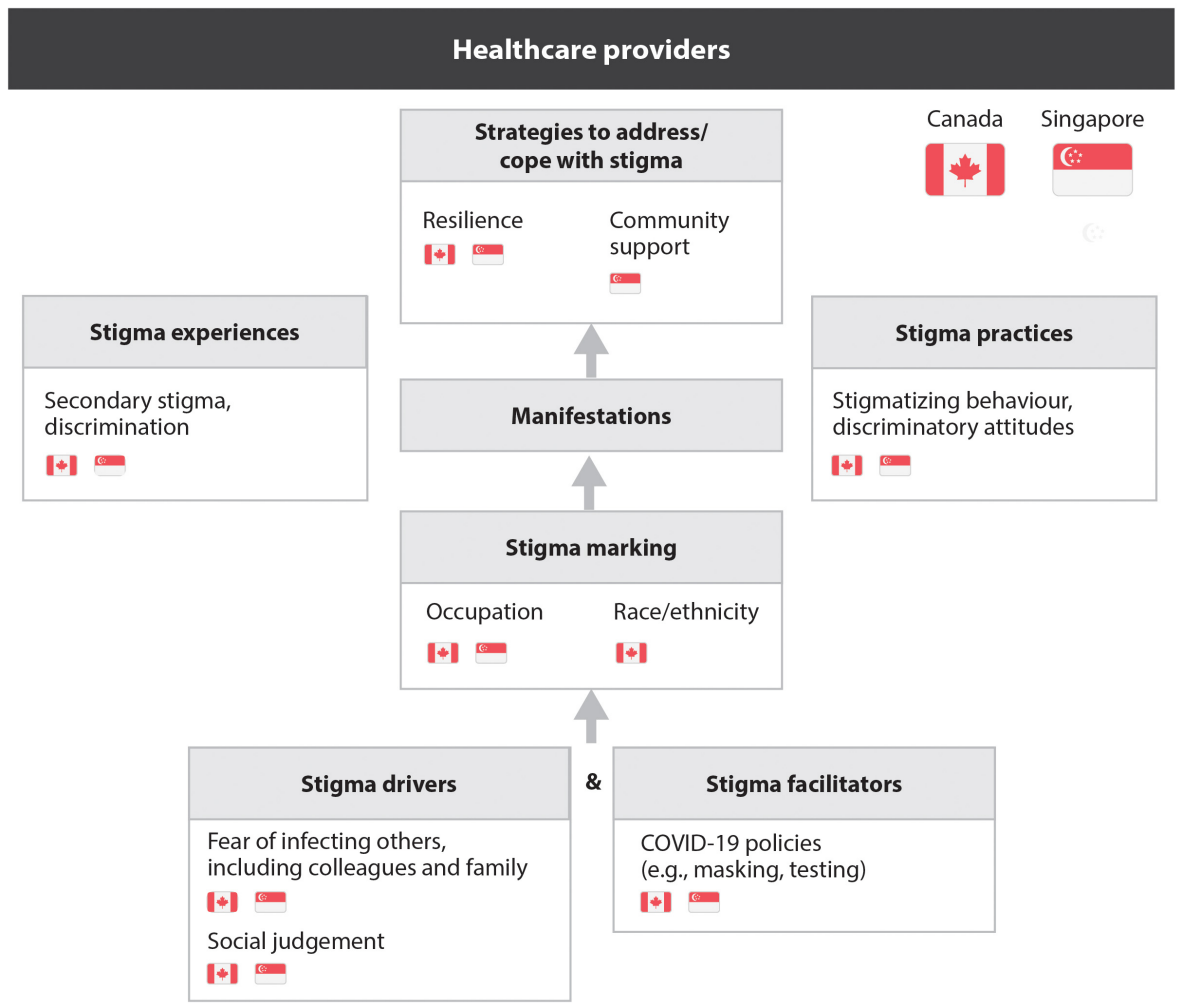
Healthcare providers' perceptions and experiences of stigma.

HCPs also faced stigma driven by fear of infection and the consequences of testing positive, such as isolation and staff shortages. Some avoided testing, which fostered distrust among colleagues. In Canada, HCPs of Chinese ethnicity experienced compounded stigma related to race and occupation, both at work and in their communities, as people feared exposure to COVID-19. Despite facing stigmatization and, in some cases, overt discrimination, HCPs relied on resilience and a sense of duty. While Singaporean HCPs noted strong community support, this was not emphasized in Canada (Figure 2).

Our findings are comparable to other North American studies which showed significant declines in hospital admissions, emergency department visits, routine care appointments, and cancer care services during the COVID-19 pandemic (29–32). Stigmatization of healthcare workers was also reported globally during the pandemic (33–39). In May 2020, a community of health and humanitarian advocates issued a declaration describing the “harassment, stigmatization and physical violence” toward healthcare workers, noting that these experiences continued despite increased shows of public support for COVID-19 responders, as noted by our sample of Singaporean HCPs (36, 39). For racialized HCPs, intersecting stigmas compounded stigma manifestations and outcomes. In our Canadian sample, HCPs who were racialized, particularly those of Chinese or East Asian ethnicity, reported intersecting racial and occupational stigmas. Other research, including from our research series on stigma during COVID-19, show an intersection of occupational and race-based stigma among Chinese and East Asian individuals in Canada, fueled by systemic cultural biases and the historical social exclusion of Asian Canadians (11, 15, 40). Previous research by our team suggests that these sentiments were reinforced by media narratives and misinformation (16).

The trends we observed during COVID-19 are consistent with those reported during other infectious disease outbreaks, including the SARS epidemic. In the seminal 2008 *Yellow Peril* report, Leung describes the intersecting race-based and occupational stigma experienced by healthcare workers of East Asian ethnicity during the 2003 SARS outbreak in Toronto (41). Health facilities that cared for Chinese-ethnicity patients were labeled “SARS facilities”; attitudes by and toward healthcare workers (particularly those of East Asian or Southeast Asian ethnicity) at these facilities were often disrespectful (41). East and Southeast Asian nurses described being the targets of anger and isolation by their colleagues; these attitudes let to systemic racism in the workplace that lasted well beyond the immediate SARS outbreak (41).

Recalling the stigma and negative impacts experienced by healthcare workers during the SARS epidemic, some organizations such as the Canadian Medical Association offered free mental health supports and resources to healthcare workers (42). Despite these efforts, healthcare workers continued to report experiences of stigmatization and harassment, well into the pandemic, with similar sentiments reported in Singapore (43). In both countries, HCPs repeatedly called for increased protections from organizations and governments (43, 44). In our study, HCPs cited their own internal resilience and desire to “do their job” to care for patients as their main source of support, and did not cite the use of external programs or supports, with the exception of some HCPs in Singapore who felt the community rallied behind them.

The need for readily available programs and messages to address occupational stigma in the face of a public health emergency is critical, given the repeated patterns observed in recent crises. The effects of occupational stigma may not be immediately felt by healthcare providers, yet can be long-lasting (41), leading to burnout (34), fatigue and other mental health challenges. This allostatic load also disproportionately impacts workers who also experience stigma related to pre-conceived biases based on their race, age, gender, and socioeconomic status (45, 46). Thus, stigmatization not only creates harm for those experiencing it, but collectively challenges the resilience of a healthcare workforce during times of emergency. A compromised health workforce impacts capacity to respond to emergencies and requires systemic interventions (47).

Use of the HSDF highlights similarities in associative stigma experienced by HCPs during COVID-19 and other infectious disease outbreaks. For instance, studies have reported parallel experiences of HCPs working in the field of HIV/AIDS compared to COVID-19 (11, 15, 40). As such, the field of HIV research can provide powerful insights on combatting stigma using evidence-based strategies (16). Systematic reviews of strategies to reduce HIV stigma in healthcare settings suggest a need to implement stigma-reducing interventions at the individual, organizational and policy levels (48, 49). At the individual level, increased awareness of what stigma is and how it impacts patient-provider interactions is warranted. Clarifying healthcare workers' knowledge of infectious disease characteristics, including corrections of falsely held beliefs on transmission, is recommended (2). At the organizational level, recommended policies include adequate personal protective equipment and infection prevention and control guidelines and policies to protect the safety of healthcare workers and patients (2). In particular, policies rooted in health equity that provide added supports to marginalized or stigmatized groups, including those disproportionally impacted by the health crises [e.g., marginalized communities in Canada living in COVID-19 hotspots (50)] are required to reduce social blame and stereotyping attributed to outbreaks (51). Notably, our review of the literature identified few studies that evaluated the impact of strategies designed specifically to address associative stigma experienced by healthcare providers (16).

Our study used a theoretical framework and in-depth interviews with HCPs in two country settings, at the height of the COVID-19 pandemic and provides a cross-cultural comparison of HCP stigma perceptions and experiences. The consistency of experiences reported across the settings magnifies the need for emergency preparedness efforts to include messaging and programs aimed at mitigating stigmatization during health emergencies. Such messages should aim to directly address sentiments of prejudice and pre-existing stereotypes; in both Canada and Singapore, a need for messages addressing racism and biases toward racialized and marginalized populations was identified. For HCPs, such messaging should include supports to deal with stigmatizing behaviors and discriminatory attitudes, including those linked to infection prevention policies (e.g., isolation protocols).

This study has limitations. In Canada, recruitment efforts were mostly centered in Ontario, particularly the Greater Toronto Area. This was reflected in our participant demographics and the sample may not be representative of the experiences held by HCPs in other regions of Canada. In both our Canadian and Singaporean samples, we noted participant experiences of intersecting stigma (e.g., occupational and racial stigma). Additional probing to describe the manifestations of intersecting stigma would have provided a richer description of this phenomenon and an exploration on how intersecting stigma led to differences in experiences or outcomes for healthcare workers. In addition, since convenience sampling was used in this study, it is possible that views in this study may represent only part of the population. For instance, in Singapore, views of non-local HCPs may differ from locals. Our study also assessed HCPs' perceptions of stigma among their patient populations, though patients utilizing the healthcare system were not interviewed. However, parallel studies conducted by our team with members of the public in Canada and Singapore provide a public perspective on fear and stigma during the COVID-19 pandemic and correlate with identified themes (15, 16, 52, 53). Moreover, the participant pool included healthcare professionals in diverse roles. Participants with patient-facing roles and occupational markers (e.g., wearing scrubs) may have had a different experience compared to other healthcare workers. Finally, our data were collected between May to October 2020 in Canada and between October 2020 to February 2021 in Singapore, which represents the first and second COVID-19 waves. It is likely that experiences and drivers of stigma evolved during the pandemic (15, 54).

## 5 Conclusion

HCPs commonly experienced associative stigma during the COVID-19 pandemic; for some participants, particularly racialized HCPs in Canada, race-based stigma intersected with occupational stigma. HCPs also noted stigmatization of patient populations, particularly those from marginalized groups. The prevalence of stigmatization of healthcare workers during a health emergency has negative impacts on individuals, but also challenges the resilience of the healthcare workforce. Future research should evaluate the impact of multi-level strategies that address stigma at both a systems and individual level. Research should also determine how strategies can be adapted to address occupation-based stigmatization and its intersections.

## Data Availability

The datasets presented in this article are not publicly available to protect the privacy of participants and adhere with research ethics boards requirements, however, data are available upon reasonable request from the corresponding author. Requests to access the datasets should be directed at: Christine Fahim, Email: christine.fahim@unityhealth.to.
