# Supplementary material for: Stigma and fear during the COVID-19 pandemic: a qualitative study on the perceptions of healthcare workers in Canada and Singapore
Source: Front Public Health. 2025 Jan 23;12:1490814. doi: 10.3389/fpubh.2024.1490814 (PMC11801013; doi:10.3389/fpubh.2024.1490814)
Supplement: Supplementary file 2 [file Data_Sheet_2.pdf]

# COVID Interview Guide for Healthcare Providers

**Study title:** Developing strategies and tools to combat misinformation, fear and stigma in the wake of COVID-19

## Section 1: General Questions

**1) Do you feel as if you have enough information on COVID-19?**

- What information do you want, but are missing?
- [If participant indicates too much information] How have you managed interpreting the overload of information?
- Is there any misinformation among healthcare providers around covid-19 that you are aware of? If so, can you describe?

**2) How have you been obtaining your information regarding how to respond to COVID-19?**

- What sources (e.g., online media, news channels, family and friends, etc.) do you look to in order to make informed treatment decisions and recommendations?
- Have any of these information sources conflicted? (e.g., hospital policy messaging versus media outlets?)
- What challenges have you experienced in terms of seeking information about COVID-19?
- What has been helpful for you in terms of obtaining relevant information about COVID-19?

**3) Could you describe your perceptions of COVID-19 versus the current reaction in the healthcare system to it?**

- Probe: Could you please speak to the various sources providing COVID-related information? Probe for thoughts/impressions of information
- Do you think your hospital/institution has communicated about COVID-19 management and mitigation strategies appropriately? Why or why not?
- How well are healthcare workers following safety standards (e.g., wearing masks, washing their hands, etc.)?
- What are your thoughts on a) local; b) provincial; c) national health policies that have been put in place to combat the spread of COVID-19?

## Section 2: Drivers & Facilitators

**1) How has COVID-19 impacted you personally?**

- How has it impacted your day-to-day life (e.g., job, health, family)?

**2) What is your greatest fear or concerns as related to COVID-19?**

  - Can you please describe any additional fears that you have experienced during the COVID-19 outbreak?

**3) What are your thoughts on the financial impact COVID-19 will have on you or on others?**

**4) What have conversations between you and your patients about the novel coronavirus or COVID-19 been like?**

- What kinds of information are your patients sharing with you? Where have they heard this information from?

**5) What have conversations between you and your coworkers about COVID-19 been like?**

- What is the information that your coworkers share with you? Where have they heard this information from?

**6) Are there aspects of your identity (e.g., race, age, gender, sexual orientation) that has impacted your willingness to seek help if you thought that you were ill with COVID-19?**

**7) Are there aspects of your patients' identity (e.g., race, age, gender, sexual orientation) that has impacted their willingness to seek help if they thought that they were ill with COVID-19?**

- Do you believe certain individuals or groups' physical health are more impacted by COVID-19 than others? If so who and why?
- Do you believe certain individuals or groups' psychosocial health are more impacted by COVID-19 than others? If so who and why?

**8) Do you think there is someone to blame for COVID-19 misinformation? If yes, who?**

• What have you seen, heard, or read that makes you feel this way?

## Section 3: Stigma Marking

**1) Do you think fear and stigma around COVID-19 has resulted in certain groups being viewed by people in a negative way?**

- If yes, what aspects of someone's identity, such as race, age, occupation, gender or sexual orientation, have been stigmatized because of COVID-19?
- How well do you think systems and supports around these groups have been able to support them in response to COVID-19 (e.g., job security, housing/rent payments, mortgage bills, access to food)?
- What are your thoughts on the accuracy of that information?
- What are your feelings towards how certain groups are targeted?

**2) Could you provide examples of misinformation on COVID-19? How are you able to distinguish between misinformation and facts?**

**3) What are your thoughts on the information on COVID-19 that the media/news (e.g., social media, news articles, TV broadcasts) share? Why do you agree/disagree with it?**

## Section 4: Stigma Experiences

**1) Have you experienced any discrimination because of COVID-19?**

- If yes, if you're comfortable, could you share what that experience was like?

**2) Have any of your patients or coworkers experienced any discrimination because of COVID-19?**

- If yes, if you're comfortable and ensuring privacy, could you share what that experience was like?

**3) [If no to the above questions] Do you fear that your patients or coworkers will experience stigma because of COVID-19?**

**4) Do you fear personally experiencing stigma because of COVID-19?**

**5) Do you have any other feedback, comments, or concerns regarding stigma, fear, or misinformation surrounding COVID-19 that we haven't discussed today?**
